# Investigating the impact of undiagnosed anxiety and depression on health and social care costs and quality of life: cross-sectional study using household health survey data

**DOI:** 10.1192/bjo.2023.596

**Published:** 2023-10-27

**Authors:** Brendan Collins, Jennifer Downing, Anna Head, Terence Comerford, Rajan Nathan, Benjamin Barr

**Affiliations:** Department of Public Health, Policy and Systems, University of Liverpool, UK; Forensic Psychiatry, Cheshire and Wirral Partnership NHS Foundation Trust, Chester, UK

**Keywords:** Anxiety or fear-related disorders, depressive disorders, health economics, epidemiology, comorbidity

## Abstract

**Background:**

There is uncertainty around the costs and health impacts of undiagnosed mental health problems.

**Aims:**

Using survey data, we aim to understand the costs and health-related quality-of-life decrements from undiagnosed anxiety/depression.

**Method:**

We analysed survey data from two waves of the North West Coast Household Health Survey, which included questions on disease, medications, and Patient Health Questionnaire 9 (PHQ-9) and Generalised Anxiety Disorder 7 (GAD-7) scores (depression and anxiety scales). People were judged as having undiagnosed anxiety/depression problems if they scored ≥5 on the PHQ-9 or GAD-7, and did not declare a mental health issue or antidepressant prescription. Linear regression for EuroQol 5-Dimension 3-Level (EQ-5D-3L) index scores, and Tweedie regression for health and social care costs, were used to estimate the impact of undiagnosed mental health problems, controlling for age, gender, deprivation and other health conditions.

**Results:**

Around 26.5% of participants had undiagnosed anxiety/depression. The presence of undiagnosed anxiety/depression was associated with reduced EQ-5D-3L index scores (0.040 lower on average) and increased costs (£250 ($310) per year on average). Using a higher cut-off score of 10 on the PHQ-9 and GAD-7 for undiagnosed anxiety/depression had similar increased costs but a greater reduction in EQ-5D-3L index scores (0.076 on average), indicating a larger impact on health-related quality of life.

**Conclusions:**

Having undiagnosed anxiety or depression increases costs and reduces health-related quality of life. Reducing stigma and increasing access to cost-effective treatments will have population health benefits.

## Common mental health problems in the UK

Common mental health problems such as mild-to-moderate depression and anxiety are estimated to affect around one in six adults in the UK.^1^ Based on data from the UK Psychiatric Morbidity Survey from 2007 and 2014, the proportion of people having some kind of treatment increased from one in four to one in three.^1^ In the UK, since the advent of selective serotonin reuptake inhibitors drugs in the 1990s, an increasing proportion of mental health problems have been treated in primary care;^2^ and since 2008, there has been a significant National Health Service (NHS) investment in psychological therapies for mild-to-moderate mental health problems.^3^ There have been studies suggesting that increasing diagnosis and treatment of common mental health problems will be cost-effective. Historically, health systems have been designed around treating individual physical health conditions, but multimorbidity (living with multiple diseases) is becoming more common, so understanding the costs of multimorbidity, including both mental and physical health problems, is important.^4^ Mental health treatment has become more compartmentalised over time, but we cannot say for certain that someone who has anxiety and depression has two ‘morbidities’. It is more likely that they have a problem that is manifest across two different diagnostic categories.

There is a lot of evidence for unmet need in mental healthcare,^5^ and that mental health interventions are cost-effective.^6^ However, there is a lack of evidence for the increased health costs of undiagnosed mental health problems in a UK context.

## Disease costs and quality of life decrements

There have been attempts to produce catalogues of costs for diseases that can be used in economic models for healthcare decision-making.^7^ There is a lack of evidence as to how mental health, age and multimorbidity interact to affect healthcare costs or produce decrements in health-related quality of life (HRQoL). There is debate about whether costs of multiple diseases are additive (e.g. the costs of coronary heart disease and cancer are added together), competitive (the cost of two diseases is less than the two added together) or synergistic/multiplicative (the cost of two diseases is more than the two costs added together).^8^ This may vary by disease and whether the diseases have common symptoms. In the absence of large data-sets, economists often treat costs for multiple diseases additively.

The objective of this study was to assess whether the association between number of concurrent diseases and healthcare costs/HRQoL was modified by the presence of undiagnosed mental health problems. We hypothesised that undiagnosed depression or anxiety may increase health costs and reduce HRQoL, relative to people with the same age, gender, deprivation and disease profile who do not have undiagnosed mental health problems.

## Method

The North-West Coast Household Health Survey was commissioned from BMG Research, with wave 1 conducted in 2015 and wave 2 in 2018. In wave 1, the survey collected data from residents of 20 disadvantaged and eight relatively advantaged neighbourhoods (based on their Index of Multiple Deprivation (IMD) quintiles) across North-West England, whereas wave 2 was focused only on disadvantaged neighbourhoods. Households were sampled with a random locational probability approach. One adult per household participated in the survey, with the ‘next birthday’ rule applied to select the participant, to reduce researcher bias. Some adults completed the survey in the first and second waves, whereas some were included in the first or second wave only. The survey interview took around 45 min and was completed by a researcher from BMG Research entering responses onto an iPad. Ethics approval for the study was obtained from the University of Liverpool (approval number RETH000836). Written informed consent was obtained from all participants. Participants gave informed consent to participate in the study before taking part. The sample size and weighting methods have been described previously.^9^ All analyses were adjusted for sampling weights. For the present paper, we included all survey participants from wave 1 and wave 2 the first time they had completed the survey. Not all individuals answered every question on the survey, and we did not impute for missing variables.

The survey included the five questions of the EuroQol 5-Dimension 3-Level (EQ-5D-3L), a commonly used measure of HRQoL. EQ-5D-3L index scores were calculated from this by using UK preference values. Costs were attached to survey resource use items, using standard reference costs from Personal Social Services Research Unit or NHS reports.^10^ All costs were adjusted to 2019 prices.

### Depression and anxiety

Depression was assessed as a score of ≥5 on the Patient Health Questionnaire 9 (PHQ-9), and anxiety by a score of ≥5 on the Generalised Anxiety Disorder 7 (GAD-7) screener. Depression or anxiety was assumed to be diagnosed when an individual scored ≥5 on the PHQ-9 and/or GAD-7 and reported a mental health issue in the past 12 months, or were being prescribed antidepressants (there was no specific question about anti-anxiety medication). If someone did not state a mental health issue or medication, their depression or anxiety was assumed to be undiagnosed on the basis of these scores. The scores of ≥5 on PHQ-9 and GAD-7 are the cut-offs for mild depression and anxiety; we also repeated the analysis with a cut-off of ≥10, which represents moderate depression and anxiety.

We looked at costs and EQ-5D-3L index scores by deprivation score and age, and by number of reported health conditions. Results were described with descriptive statistics. Costs included emergency department and minor injury unit attendances, ambulance conveyances, hospital admissions, general practitioner (GP) visits, home visits from a GP or nurse, and allied health professional (health visitor, occupational therapy, dietician, psychologist, counsellor, podiatrist) visits. Social care costs were limited to social worker contacts only. See Supplementary Appendix 1 available at https://doi.org/10.1192/bjo.2023.596 for the full list of costs and sources, which has been described previously.^11^

**Fig. 1:**
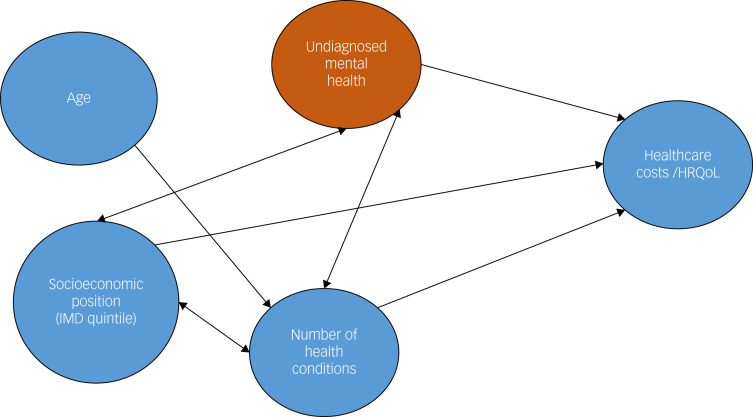
Hypothesised relationship between age, socioeconomic position, number of diseases, undiagnosed mental health problems and healthcare costs, and health-related quality of life (HRQoL). IMD, Index of Multiple Deprivation.

Figure 1 shows the hypothesised relationship between the variables we modelled, except for gender, where we did not have a prespecified hypothesis of its effect. The relationship between multiple diseases and undiagnosed mental health problems is assumed to be bidirectional.^12^ Linear regression of EQ-5D-3L index scores was carried out with age (years), gender, area-level IMD quintile and number of reported health conditions (from zero to 16) as independent variables. The possible health conditions were cancer, diabetes, epilepsy/fits, migraine or other frequent headache, dementia/Alzheimer's disease, any mental health issue, cataracts/eyesight problems (including those corrected with glasses or contacts), ear/hearing problems (including those corrected with a hearing aid), stroke, heart attack/angina, high blood pressure, bronchitis/emphysema, asthma, allergies, stomach ulcer or other digestive problems, liver problems, bowel/colon problems, bladder problems/incontinences, arthritis, gout, skin problems and other (other was treated as one additional condition in the regression). We carried out this regression with and without undiagnosed mental health problems to see if this had a moderating effect, which we judged by whether it changed the overall measure of fit (*R*^2^). This was to test whether undiagnosed depression or anxiety had an effect on any relationship between number of health conditions and EQ-5D-3L index score.

For costs, we followed the same process, but we used Tweedie regression, which is well-designed for cost data that show a skewed distribution and have a large proportion of zeros. Tweedie regression outperforms two part regressions for these kind of data, as well as being easier to interpret, whereas in a two-part regression, the variables that predict the presence of costs are often the same variables that predict the magnitude of costs.^13^ In our cost data, around 24% of weighted participants had zero health and social care costs. For the addition of undiagnosed mental health problems to the Tweedie regression formula, we judged whether the relationship was moderated by whether the Akaike information criterion (AIC) and Bayesian information criterion (BIC) were reduced. A lower AIC and BIC indicate that a model has a better goodness-of-fit, factoring in the increased number of predictor variables.

Data were analysed with the statistical software package SPSS version 27 for Windows.

## Results

Across the sample (*N* = 6790), the average EQ-5D-3L index score was 0.833 (s.d. 0.280), whereas the average health and social care cost over the past 12 months was £885 (s.d. £3840; $1097, s.d. $4762). For people with undiagnosed mental health problems (*n* = 1797), the average EQ-5D-3L index score was 0.793 (s.d. 0.295) and average costs were £1137 (s.d. £5070; $1410, s.d. $6287). For people without undiagnosed mental health problems (*n* = 4992), the average EQ-5D-3L index score was 0.848 (s.d. 0.272) and average costs were £794 (s.d. £3282; $985, s.d. $4070) (see Table 1).

**Table 1:** Descriptive statistics for individuals with and without undiagnosed mental health problems

	Without undiagnosed mental health problems			With undiagnosed mental health problems			All		
	Number	Mean	s.d.	Number	Mean	s.d.	Number	Mean	s.d.
Gender (% male)	4992 (50.5%)			1797 (47.6%)			6790 (49.7%)		
Age	4464	46.3	18.6	1636	46.6	19.2	6099	46.4	18.8
Education level (1–3)	3231	1.8	0.5	1230	1.8	0.5	4461	1.8	0.5
IMD quintile (1–5)	4926	4.39	1.121	1749	4.5	1.0	6675	4.4	1.1
Number of conditions	4992	1.2	1.7	1797	1.3	1.7	6790	1.2	1.7
EQ-5D-3L index score	4985	0.848	0.272	1795	0.793	0.295	6780	0.833	0.280
GAD-7 score (out of 21)	4918	2.3	4.5	1797	6.2	4.7	6716	3.3	4.8
PHQ-9 score (out of 27)	4820	2.9	5.1	1797	8.5	4.9	6617	4.4	5.6
Total health and social care costs	4992	£794	£3282	1797	£1137	£5070	6790	£885	£3840

Number is the re-weighted number of people who answered each question, and not all participants answered all questions. Some totals for the two categories of with and without undiagnosed mental health problems do not add up because of rounding, as a weighting factor is applied to each individual in the survey data. IMD, Index of Multiple Deprivation; EQ-5D-3L, EuroQol 5-Dimension 3-Level; GAD-7, Generalised Anxiety Disorder 7; PHQ-9, Patient Health Questionnaire 9.

**Fig. 2:**
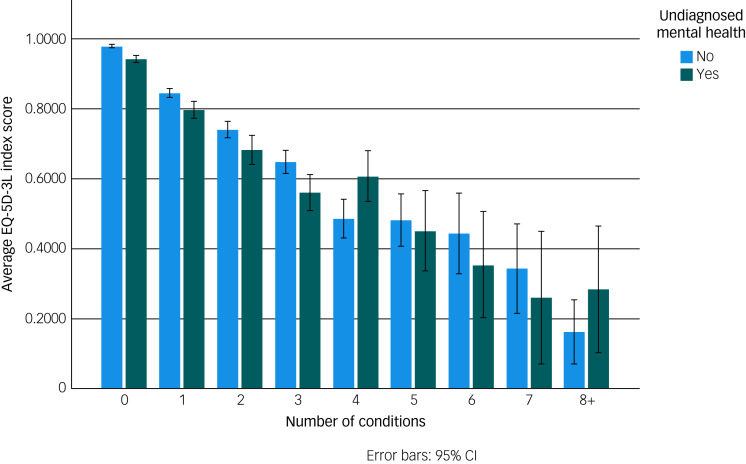
Average EQ-5D-3L index scores by number of conditions and whether an individual has undiagnosed mental health problem. EQ-5D-3L, EuroQol 5-Dimension 3-Level.

**Fig. 3:**
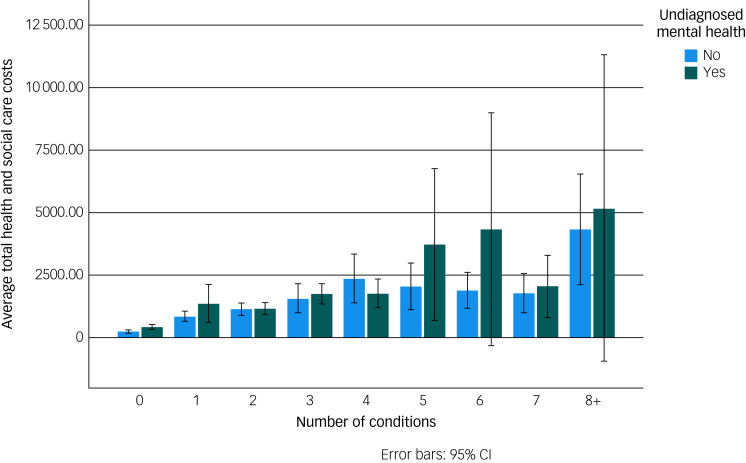
Average health and social care costs by number of conditions and whether an individual has undiagnosed mental health problem.

There was a pronounced relationship between increasing number of health conditions and healthcare costs, and a strong relationship between increasing number of health conditions and diminished HRQoL, as measured with EQ-5D-3L index scores (Figs 2 and 3). Having an undiagnosed mental health condition had a modifying effect in terms of increasing healthcare costs and decreasing HRQoL. In a linear regression of EQ-5D-3L index score, the addition of undiagnosed mental health problems increased the adjusted *R*^2^ value from 0.378 to 0.382. The coefficient for undiagnosed mental health problems was −0.040. Apart from gender, all predictor variables were significant at a 0.001 level.

Overall for those individuals within the survey, we get an equation where:

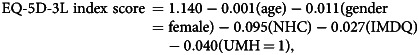
where NHC is number of health conditions, IMDQ is IMD quintile and UMH is undiagnosed mental health problems.

So, for example, for a 60-year-old woman with three health conditions, living in IMD quintile 5, with undiagnosed mental health problems, the predicted EQ-5D-3L index score would be 0.609. This type of equation may be useful in estimating the quality-adjusted life-years (QALYs) lost or gained from mental health interventions.

For healthcare costs, we used Tweedie regression, with health and social care costs per person in the past 12 months as the output variable. The addition of undiagnosed mental health problems to the regression equation reduced the AIC from 86 765 to 86 627, and reduced the BIC from 86 805 to 86 673, meaning that this inclusion produced a better-fitting regression model. In the final model, all predictors were significant at the 0.001 level, apart from age (*P* = 0.294). The coefficient for undiagnosed mental health problems was 250, meaning that having undiagnosed mental health problems increased costs by around £250 ($310), whereas each health condition increased average costs by around £505 ($626). Of note, the coefficient for age in this equation is negative and not statistically significant, meaning that once other predictors of healthcare costs like multimorbidity were controlled for, increasing age did not have a significant effect on costs. The coefficient for IMD quintile was also negative, which was somewhat counterintuitive, indicating that the effects of deprivation in the sample were mainly mediated by the presence of long-term conditions – as the coefficient for IMD quintile was positive when long term conditions and undiagnosed mental health was taken out of the equation (see Supplementary Appendix 1).

Overall, we get a regression equation where:

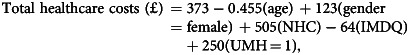
where NHC is number of health conditions, IMDQ is IMD quintile and UMH is undiagnosed mental health problems.

For example, for a 50-year-old man with two health conditions, living in IMD quintile 3, with undiagnosed mental health problems, average annual health and social care costs would be:
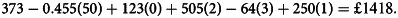


Using a GBP to USD conversion of 1.24 (correct as of April 2023), this equates to around $1758.

As an additional sensitivity analysis, we used a higher cut-off (score of 10 instead of 5, indicating moderate anxiety or depression) on the GAD-7 and PHQ-9 for the definition of mental health problems, which meant that 686 people in the data (10.1% of the valid sample) were classed as having undiagnosed mental health problems instead of 1797 (26.5%) people. This changed the coefficient for EQ-5D-3L index score from −0.040 to −0.076, indicating a greater health loss for undiagnosed mental health problems when using this higher cut-off; the coefficient for health and social care costs was almost unchanged, at around £250 ($310) additional costs per year.

## Discussion

We found that number of conditions was a significant predictor of increased health and social care costs, and reduced HRQoL. Furthermore, we found that undiagnosed mental health problems had a moderating effect on the relationship between age, deprivation, number of conditions as independent variables and costs and HRQoL as dependent variables, whereby costs were increased, and HRQoL was further reduced when an individual had an undiagnosed mental health problem. For both costs and HRQoL, the effect of undiagnosed mental health problems was about 50% of the effect of having an additional disease. The coefficient for undiagnosed mental health problems on EQ-5D-3L index score was −0.040, which is a significant effect on overall HRQoL. As a comparison, this is greater than the coefficient for having atrial fibrillation from the USA-based Medical Expenditure Panel Survey (−0.0384).^14^ Each year lived with this utility deficit equates to 0.040 QALYs lost, which, if valued at £30 000 per QALY, would be worth £1200, or if valued at $100 000, would be $4000.

The significance of our research is that it quantifies the additional cost and health losses attributable to undiagnosed anxiety and depression, and contextualises it in terms of how it relates to the costs and health losses from other comorbidities.

### For policy makers and modellers

These results suggest that, for the survey sample in North-West England, multimorbidity is a much bigger driver of HRQoL and healthcare costs than age. Once number of diseases, deprivation and undiagnosed mental health problems are controlled for, the impact of age on health and social care costs and HRQoL in our regression becomes close to zero. The costs and QALY losses from undiagnosed mental health conditions were roughly 40–50% of the costs of having an additional diagnosed condition (£250 ($310) for undiagnosed mental health, £505 ($626) for an additional condition; 0.040 QALY loss per year of undiagnosed mental health, 0.095 QALY loss for an additional health condition). However, based on a higher cut-off on the GAD-7 and/or PHQ-9, the QALY loss for undiagnosed mental health was closer to having an additional health condition, at 0.076 per year.

The EQ-5D-3L index score results from this study may prove useful for health economic models, although there may be other sources that are more representative of the general population, such as the Health Survey for England or GP Survey. The Health Outcomes Data Repository (HODAR) and the EQ-5D Medical Expenditure Panel Survey (MEPS) catalogue, which are often used, are also limited, as the MEPS data originates from the USA and the HODAR data concerns people discharged from hospital in Cardiff.

### Comparison with other studies

Overall average health and social care costs across the sample were around £1000 in the past 12 months, which is less than the true figure of closer to £3000, based on estimates for the UK.^15^ The majority of UK healthcare costs are for hospital admissions, which are less common and more costly, and we have used a relatively crude average for hospital activity in this study as we do not have a breakdown of why people were admitted. Also resource use in the survey is based on recall, which can be unreliable.^16^

A systematic review published in 2007 found that estimated costs of depression varied considerably, as did methods of estimating costs.^17^ A study published by the King's Fund in 2008^18^ estimated the average cost per case of depression in England as around £1354 in 2007 prices, or around £1684 in 2019 prices; and the cost per case of anxiety as around £544 in 2007, or around £676 in 2019 prices. Our analysis did not separate out anxiety and depression, but if the average cost was around £1000, then undiagnosed cases may have around a quarter of the average cost, at around £250.

In terms of costs of undiagnosed diseases, to our knowledge, this is the only study looking at anxiety and depression, but there have been studies of other diseases. A 2007 USA study^19^ found that costs of undiagnosed diabetes were higher than similar individuals without undiagnosed diabetes, but lower than individuals with diagnosed diabetes, similar to our findings about mental health costs. A similar pattern was observed in USA studies of undiagnosed atrial fibrillation costs and chronic obstructive pulmonary disease costs.^20,21^

There is a literature around delayed diagnosis that is similar but not quite the same as undiagnosed, as many of the participants from the present study may never be diagnosed, or some may recover without treatment. For instance, a study of delayed diagnosis of bipolar disorder in California, USA, found that costs were higher in people who had delayed diagnosis.^22^ There has been an increased focus on delayed diagnosis since the pandemic, with studies looking at delayed diagnosis of long-term conditions in primary care in Wales,^23^ and delayed cancer diagnoses in the USA.^24^

### Strengths and weaknesses

The sample areas were not representative of England, or even North-West England, but most areas were deprived, which meant that there was a high disease prevalence. In this study, we talk about undiagnosed depression and anxiety, but we do not know what proportion of this is definitely undiagnosed – it may be unreported or untreated, rather than undiagnosed.

### Future work

Future work could combine the EQ-5D-3L data from this study with mortality data to estimate quality-adjusted life expectancy across the population.

We would encourage studies to consider how unreported mental health problems are related to future healthcare costs and QALYs. Trials of the cost-effectiveness of increasing diagnosis and treatment of common mental health problems across the population would also be productive. Further, one study found that collaborative care for people with physical–mental multimorbidity is effective and cost-effective.^25^

This study demonstrates that undiagnosed mental health problems exacerbate existing multimorbidity by further increasing health and social care costs, and further reducing HRQoL. Efforts need to be increased to diagnose and treat mental health problems like anxiety and depression.

## Supporting information

Collins et al. supplementary material Collins et al. supplementary material

## Data Availability

The North-West Coast Household Health Survey data is available to researchers on signing a data use agreement.
