# Investigating the impact of undiagnosed anxiety and depression on health and social care costs and quality of life: cross-sectional study using household health survey data – CORRIGENDUM

**DOI:** 10.1192/bjo.2023.630

**Published:** 2023-12-18

**Authors:** Brendan Collins, Jennifer Downing, Anna Head, Terence Comerford, Rajan Nathan, Benjamin Barr

**Keywords:** Anxiety or fear-related disorders, depressive disorders, health economics, epidemiology, comorbidity

This article was originally published with author Terence Comerford's surname spelled incorrectly. The error has been corrected and the online PDF and HTML versions updated.
