# Supplementary material for: Investigating the impact of undiagnosed anxiety and depression on health and social care costs and quality of life: cross-sectional study using household health survey data
Source: BJPsych Open. 2023 Oct 27;9(6):e201. doi: 10.1192/bjo.2023.596 (PMC10753950; doi:10.1192/bjo.2023.596)
Supplement: Collins et al. supplementary material [file S2056472423005963sup001.pdf]

Are health issues exacerbated by undiagnosed mental health problems? Cross-sectional study using a household health survey to estimate healthcare costs and quality of life related to multimorbidity.

Appendices. Full SPSS output files and additional figures.

Figure A1. Costs by number of conditions.

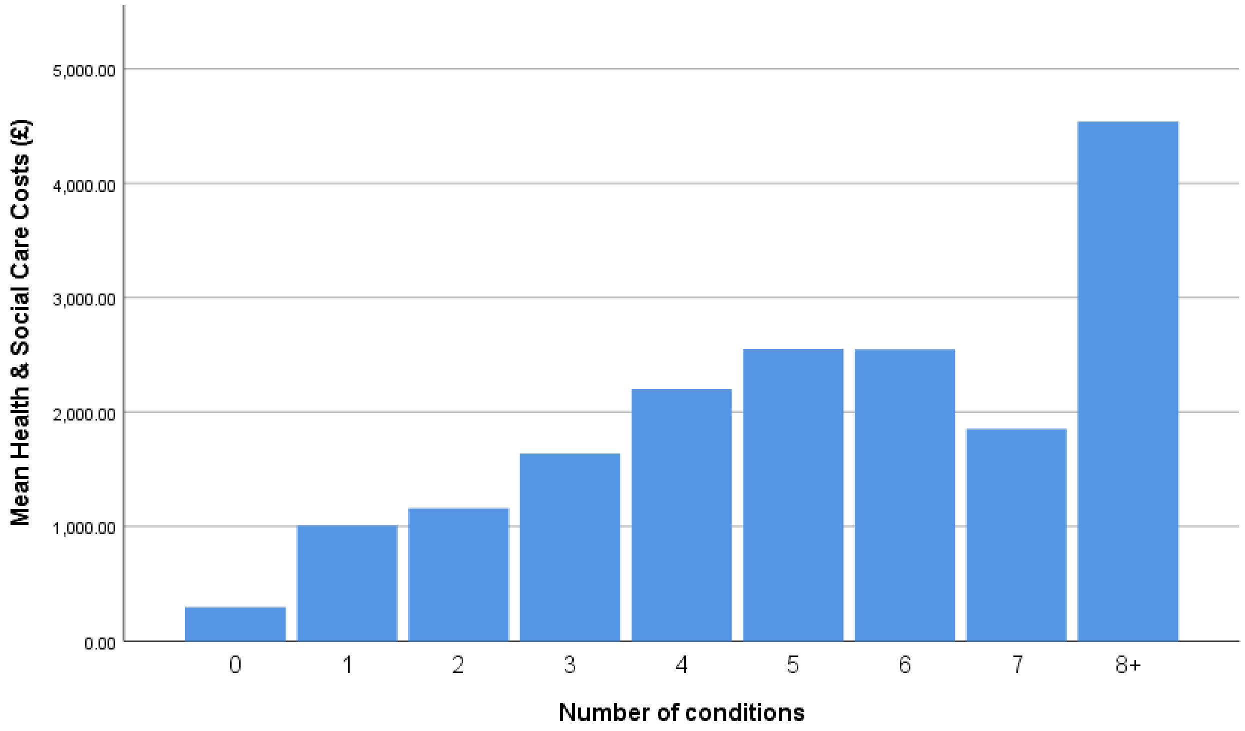

Figure A2. EQ-5D index scores by number of conditions

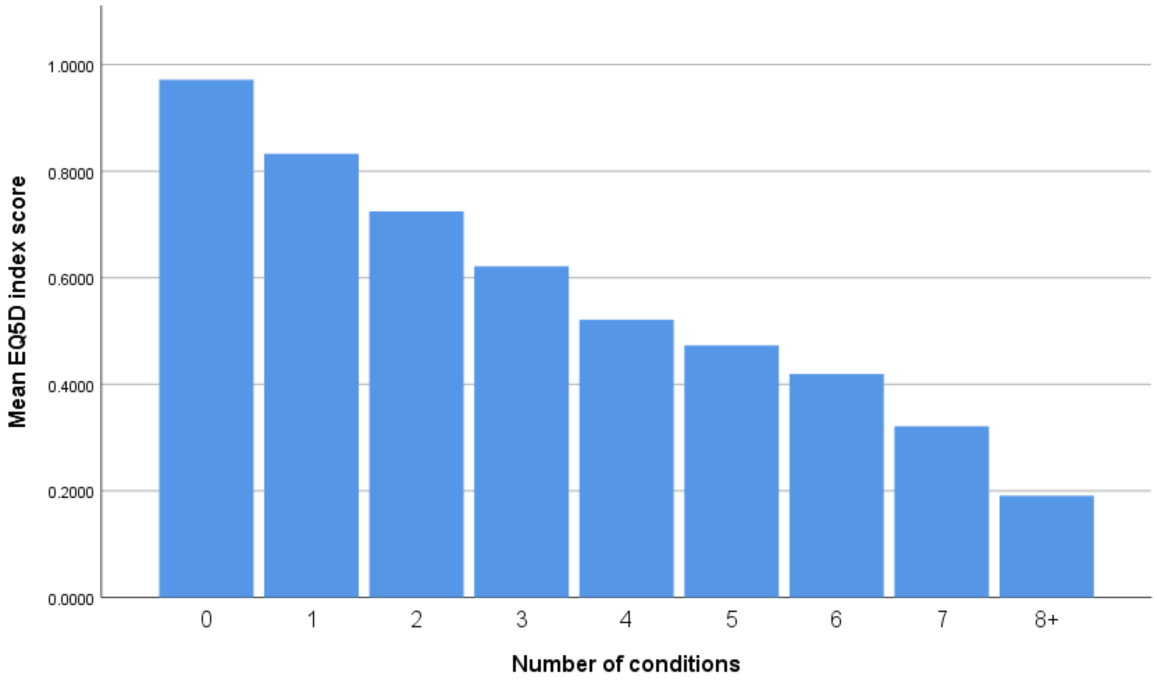

Figure A3. Costs by age.

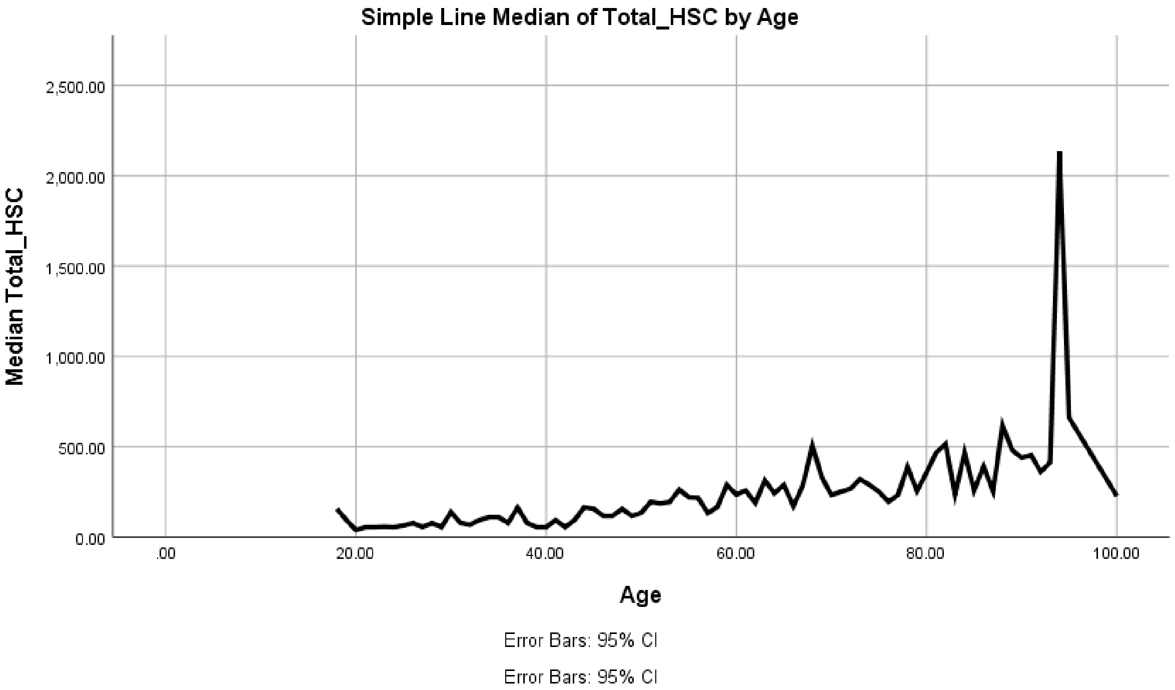

.

Figure A4. EQ-5D index scores by age.

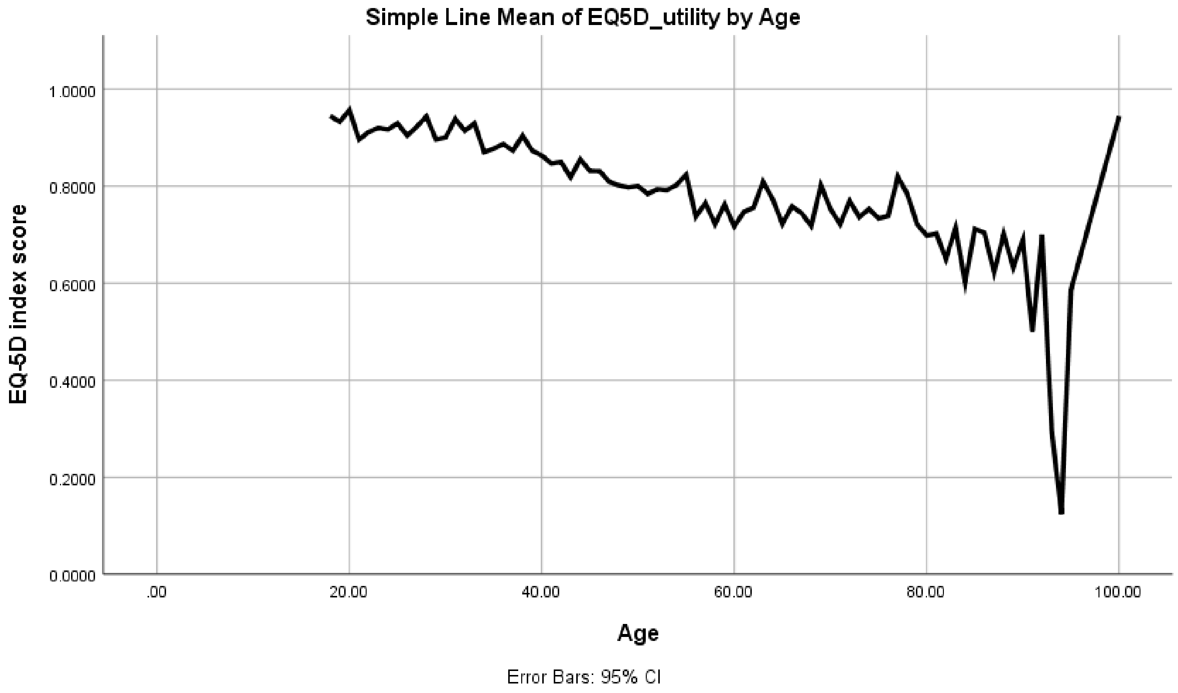

1. HEALTH&SOCIAL CARE COSTS REGRESSION WITHOUT UNDIAGNOSED MENTAL HEALTH

```
* Generalized Linear Models.
GENLIN USE_Total_health_social_care_costs WITH Age Gender_1st_participant N_conditions quint_imd
  /MODEL Age Gender_1st_participant N_conditions quint_imd INTERCEPT=YES SCALEWEIGHT=WEIGHT5
 DISTRIBUTION=TWEEDIE(1.5) LINK=IDENTITY
  /CRITERIA METHOD=FISHER SCALE=MLE COVB=MODEL MAXITERATIONS=50000 MAXSTEPHALVING=10
    PCONVERGE=1E-006(ABSOLUTE) SINGULAR=1E-012 ANALYSISTYPE=3(WALD) CILEVEL=95 CITYPE=WALD
    LIKELIHOOD=FULL
  /MISSING CLASSMISSING=EXCLUDE
  /PRINT CPS DESCRIPTIVES MODELINFO FIT SUMMARY SOLUTION.
```

Generalized Linear Models

Notes

**Output Created**	11-DEC-2019 15:50:50
**Comments**	

**Input**	**Data**	**M:\HHS work\wave12_withgeo_CG.sav**
	**Active Dataset**	**DataSet1**
	**Filter**	**New_or_repeat=1 (FILTER)**
	**Weight**	**<none>**
	**Split File**	**<none>**
	**N of Rows in Working Data File**	**6675**
**Missing Value Handling**	**Definition of Missing**	**User-defined missing values for factor, subject and within-subject variables are treated as missing.**
	**Cases Used**	**Statistics are based on cases with valid data for all variables in the model.**
**Weight Handling**		**not applicable**

**Syntax**		**GENLIN USE_Total_health_social_care_costs WITH Age Gender_1st_participant N_conditions quint_imd**<br>**/MODEL Age Gender_1st_participant N_conditions quint_imd INTERCEPT=YES SCALEWEIGHT=WEIGHT5**<br>**DISTRIBUTION=TWEEDIE(1.5) LINK=IDENTITY**<br>**/CRITERIA METHOD=FISHER SCALE=MLE COVB=MODEL MAXITERATIONS=50000 MAXSTEPHALVING=10**<br>**PCONVERGE=1E-006(ABSOLUTE) SINGULAR=1E-012 ANALYSISTYPE=3(WALD) CILEVEL=95 CITYPE=WALD**<br>**LIKELIHOOD=FULL**<br>**/MISSING CLASSMISSING=EXCLUDE**<br>**/PRINT CPS DESCRIPTIVES MODELINFO FIT SUMMARY SOLUTION.**
**Resources**	**Processor Time**	**00:00:02.36**

Elapsed Time	00:00:02.35

Warnings

The maximum number of step-halvings was reached but the log-likelihood value cannot be further improved. Output for the last iteration is displayed.
The GENLIN procedure continues despite the above warning(s). Subsequent results shown are based on the last iteration. Validity of the model fit is uncertain.

Model Information

Dependent Variable	Total_HSC
Probability Distribution	Tweedie (1.5)
Link Function	Identity
Scale Weight Variable	WEIGHT5

Case Processing Summary

	N	Percent
Included	5998	89.9%
Excluded	677	10.1%

Total	6675	100.0%

Continuous Variable Information

		N	Minimum	Maximum	Mean	Std. Deviation
Dependent Variable	Total_HSC	5998	.00	203754.00	1003.1209	4290.19466
Covariate	Age	5998	18.00	100.00	49.0027	19.29905
	Gender_1st_participant	5998	1.00	2.00	1.5522	.49731
	N_conditions	5998	0	16	1.46	1.900
	quint_imd	5998	1	5	4.43	1.071
Scale Weight	WEIGHT5	5998	0	10	1.0169	.75133

Goodness of Fit[a]

	Value	df	Value/df
Deviance	457518.484	5993	76.342
Scaled Deviance	10611.519	5993	
Pearson Chi-Square	3475498.128	5993	579.926
Scaled Pearson Chi-Square	80609.453	5993	
Log Likelihood[b]	-43376.305		

Akaike's Information Criterion (AIC)	86764.611		
Finite Sample Corrected AIC (AICC)	86764.625		
Bayesian Information Criterion (BIC)	86804.806		
Consistent AIC (CAIC)	86810.806		

Dependent Variable: Total_HSC

Model: (Intercept), Age, Gender_1st_participant, N_conditions, quint_imd

a. Information criteria are in smaller-is-better form.

b. The full log likelihood function is displayed and used in computing information criteria.

Omnibus Test[a]

Likelihood Ratio Chi-Square	df	Sig.
2291.749	4	.000

Dependent Variable: Total_HSC

Model: (Intercept), Age, Gender_1st_participant, N_conditions, quint_imd

a. Compares the fitted model against the intercept-only model.

Tests of Model Effects

Source	Type III Wald Chi-Square	df	Sig.
(Intercept)	68.181	1	.000
Age	8.847	1	.003
Gender_1st_participant	50.048	1	.000
N_conditions	1430.535	1	.000
quint_imd	39.522	1	.000

Dependent Variable: Total_HSC

Model: (Intercept), Age, Gender_1st_participant, N_conditions, quint_imd

Parameter Estimates

			95% Wald Confidence Interval		Hypothesis Test		
Parameter	B	Std. Error	Lower	Upper	Wald Chi-Square	df	Sig.
(Intercept)	420.044	50.8702	320.341	519.748	68.181	1	.000
Age	-1.407	.4731	-2.334	-.480	8.847	1	.003
Gender_1st_participant	121.686	17.2008	87.973	155.399	50.048	1	.000
N_conditions	519.127	13.7254	492.226	546.028	1430.535	1	.000
quint_imd	-52.568	8.3618	-68.956	-36.179	39.522	1	.000

(Scale)	43.115[a]	.5096	42.128	44.126			

Dependent Variable: Total_HSC

Model: (Intercept), Age, Gender_1st_participant, N_conditions, quint_imd

a. Maximum likelihood estimate.

HEALTH&SOCIAL CARE COSTS REGRESSION MODEL WITH UNDIAGNOSED MENTAL HEALTH

```
* Generalized Linear Models.
GENLIN USE_Total_health_social_care_costs WITH Age Gender_1st_participant N_conditions quint_imd
    undiagnosed_MH_use
  /MODEL Age Gender_1st_participant N_conditions quint_imd undiagnosed_MH_use INTERCEPT=YES
    SCALEWEIGHT=WEIGHT5
 DISTRIBUTION=TWEEDIE(1.5) LINK=IDENTITY
  /CRITERIA METHOD=FISHER SCALE=MLE COVB=MODEL MAXITERATIONS=50000 MAXSTEPHALVING=10
    PCONVERGE=1E-006(ABSOLUTE) SINGULAR=1E-012 ANALYSISTYPE=3(WALD) CILEVEL=95 CITYPE=WALD
    LIKELIHOOD=FULL
  /MISSING CLASSMISSING=EXCLUDE
  /PRINT CPS DESCRIPTIVES MODELINFO FIT SUMMARY SOLUTION.
```

Generalized Linear Models

Notes

**Output Created**		**11-DEC-2019 15:51:46**
**Comments**		
**Input**	**Data**	**M:\HHS work\wave12_withgeo_CG.sav**
	**Active Dataset**	**DataSet1**
	**Filter**	**New_or_repeat=1 (FILTER)**
	**Weight**	**<none>**
	**Split File**	**<none>**
	**N of Rows in Working Data File**	**6675**
**Missing Value Handling**	**Definition of Missing**	**User-defined missing values for factor, subject and within-subject variables are treated as missing.**
	**Cases Used**	**Statistics are based on cases with valid data for all variables in the model.**
**Weight Handling**		**not applicable**

**Syntax**	**GENLIN USE_Total_health_social_care_costs WITH Age Gender_1st_participant N_conditions quint_imd**<br><br>**undiagnosed_MH_use**<br><br>**/MODEL Age Gender_1st_participant N_conditions quint_imd undiagnosed_MH_use INTERCEPT=YES**<br><br>**SCALEWEIGHT=WEIGHT5**<br><br>**DISTRIBUTION=TWEEDIE(1.5) LINK=IDENTITY**<br><br>**/CRITERIA METHOD=FISHER SCALE=MLE COVB=MODEL MAXITERATIONS=50000 MAXSTEPHALVING=10**<br><br>**PCONVERGE=1E-006(ABSOLUTE) SINGULAR=1E-012 ANALYSISTYPE=3(WALD) CILEVEL=95 CITYPE=WALD**<br><br>**LIKELIHOOD=FULL**<br><br>**/MISSING CLASSMISSING=EXCLUDE**<br><br>**/PRINT CPS DESCRIPTIVES MODELINFO FIT SUMMARY SOLUTION.**

Resources	Processor Time	00:00:02.30
	Elapsed Time	00:00:02.29

Warnings

The maximum number of step-halvings was reached but the log-likelihood value cannot be further improved. Output for the last iteration is displayed.
The GENLIN procedure continues despite the above warning(s). Subsequent results shown are based on the last iteration. Validity of the model fit is uncertain.

Model Information

Dependent Variable	Total_HSC
Probability Distribution	Tweedie (1.5)
Link Function	Identity
Scale Weight Variable	WEIGHT5

Case Processing Summary

	N	Percent
Included	5998	89.9%

Excluded	677	10.1%
Total	6675	100.0%

Continuous Variable Information

		N	Minimum	Maximum	Mean	Std. Deviation
Dependent Variable	Total_HSC	5998	.00	203754.00	1003.1209	4290.19466
Covariate	Age	5998	18.00	100.00	49.0027	19.29905
	Gender_1st_participant	5998	1.00	2.00	1.5522	.49731
	N_conditions	5998	0	16	1.46	1.900
	quint_imd	5998	1	5	4.43	1.071
	undiagnosed_MH_use	5998	.00	1.00	.2656	.44168
Scale Weight	WEIGHT5	5998	0	10	1.0169	.75133

Goodness of Fit[a]

	Value	df	Value/df
Deviance	451509.842	5992	75.352
Scaled Deviance	10575.248	5992	
Pearson Chi-Square	3264449.300	5992	544.801
Scaled Pearson Chi-Square	76459.820	5992	

**Log Likelihood[b]**	**-43306.282**		
**Akaike's Information Criterion (AIC)**	**86626.565**		
**Finite Sample Corrected AIC (AICC)**	**86626.584**		
**Bayesian Information Criterion (BIC)**	**86673.459**		
**Consistent AIC (CAIC)**	**86680.459**		

Dependent Variable: Total_HSC

Model: (Intercept), Age, Gender_1st_participant, N_conditions, quint_imd, undiagnosed_MH_use

a. Information criteria are in smaller-is-better form.

b. The full log likelihood function is displayed and used in computing information criteria.

Omnibus Test[a]

Likelihood Ratio Chi-Square	df	Sig.
**2431.795**	**5**	**.000**

Dependent Variable: Total_HSC

Model: (Intercept), Age, Gender_1st_participant, N_conditions, quint_imd, undiagnosed_MH_use

a. Compares the fitted model against the intercept-only model.

Tests of Model Effects

**Source**	**Type III Wald Chi-Square**	**df**	**Sig.**
**(Intercept)**	**58.364**	**1**	**.000**
**Age**	**1.102**	**1**	**.294**
**Gender_1st_participant**	**58.557**	**1**	**.000**
**N_conditions**	**1393.999**	**1**	**.000**
**quint_imd**	**62.029**	**1**	**.000**
**undiagnosed_MH_use**	**108.275**	**1**	**.000**

Dependent Variable: Total_HSC

Model: (Intercept), Age, Gender_1st_participant, N_conditions, quint_imd, undiagnosed_MH_use

Parameter Estimates

			**95% Wald Confidence Interval**		**Hypothesis Test**		
**Parameter**	**B**	**Std. Error**	**Lower**	**Upper**	**Wald Chi-Square**	**df**	**Sig.**
**(Intercept)**	**372.591**	**48.7710**	**277.002**	**468.181**	**58.364**	**1**	**.000**

**Age**	-.455	.4337	-1.305	.395	1.102	1	.294
**Gender_1st_participant**	122.914	16.0624	91.432	154.396	58.557	1	.000
**N_conditions**	505.131	13.5292	478.615	531.648	1393.999	1	.000
**quint_imd**	-64.277	8.1613	-80.273	-48.281	62.029	1	.000
**undiagnosed_MH_use**	249.953	24.0212	202.872	297.033	108.275	1	.000
**(Scale)**	42.695[a]	.5053	41.716	43.697			

Dependent Variable: Total_HSC

Model: (Intercept), Age, Gender_1st_participant, N_conditions, quint_imd, undiagnosed_MH_use

a. Maximum likelihood estimate.

2. REGRESSION OF COSTS WITH MODERATE CUTOFF FOR GAD7 AND PHW9

Generalized Linear Models

Notes

**Output Created**		10-JAN-2023 15:50:26
**Comments**		
**Input**	**Data**	C:\Users\brenc\Documents\work\hhs paper\wave12_withgeo_CG from M drive.sav

	**Active Dataset**	**DataSet1**
	**Filter**	**New_or_repeat = 1 (FILTER)**
	**Weight**	**<none>**
	**Split File**	**<none>**
	**N of Rows in Working Data File**	**6675**
**Missing Value Handling**	**Definition of Missing**	**User-defined missing values for factor, subject and within-subject variables are treated as missing.**
	**Cases Used**	**Statistics are based on cases with valid data for all variables in the model.**
**Weight Handling**		**not applicable**

**Syntax**	**GENLIN USE_Total_health_social_care_costs WITH Age Gender_1st_participant N_conditions quint_imd<br><br>undiagnosed_based_on_moderat<br><br>/MODEL Age Gender_1st_participant N_conditions quint_imd undiagnosed_based_on_moderat INTERCEPT=YES<br><br>SCALEWEIGHT=WEIGHT5<br><br>DISTRIBUTION=TWEEDIE(1.5) LINK=IDENTITY<br><br>/CRITERIA METHOD=FISHER SCALE=MLE COVB=MODEL MAXITERATIONS=50000 MAXSTEPHALVING=10<br><br>PCONVERGE=1E-006(ABSOLUTE) SINGULAR=1E-012 ANALYSISTYPE=3(WALD) CILEVEL=95 CITYPE=WALD<br><br>LIKELIHOOD=FULL<br><br>/MISSING CLASSMISSING=EXCLUDE<br><br>/PRINT CPS DESCRIPTIVES MODELINFO FIT**

**Resources**	**Processor Time**	**00:00:02.14**
	**Elapsed Time**	**00:00:02.14**

Model Information

**Dependent Variable**	**Total_HSC**
**Probability Distribution**	**Tweedie (1.5)**
**Link Function**	**Identity**
**Scale Weight Variable**	**WEIGHT5**

Case Processing Summary

	N	Percent
**Included**	**5998**	**89.9%**
**Excluded**	**677**	**10.1%**
**Total**	**6675**	**100.0%**

Continuous Variable Information

		N	Minimum	Maximum	Mean	Std. Deviation
**Dependent Variable**	**Total_HSC**	**5998**	**.00**	**203754.00**	**1003.1209**	**4290.19466**

Covariate	Age	5998	18.00	100.00	49.0027	19.29905
	Gender_1st_participant	5998	1.00	2.00	1.5522	.49731
	N_conditions	5998	0	16	1.46	1.900
	quint_imd	5998	1	5	4.43	1.071
	undiagnosed_based_on_moderat	5998	.00	1.00	.1047	.30619
Scale Weight	WEIGHT5	5998	0	10	1.0169	.75133

Goodness of Fit[a]

	Value	df	Value/df
Deviance	455197.009	5992	75.967
Scaled Deviance	10597.555	5992	
Pearson Chi-Square	3673833.680	5992	613.123
Scaled Pearson Chi-Square	85531.439	5992	
Log Likelihood[b]	-43349.333		
Akaike's Information Criterion (AIC)	86712.666		
Finite Sample Corrected AIC (AICC)	86712.684		
Bayesian Information Criterion (BIC)	86759.560		

**Consistent AIC (CAIC)**	**86766.560**		

Dependent Variable: Total_HSC

Model: (Intercept), Age, Gender_1st_participant, N_conditions, quint_imd, undiagnosed_based_on_moderat

a. Information criteria are in smaller-is-better form.

b. The full log likelihood function is displayed and used in computing information criteria.

Omnibus Test[a]

Likelihood Ratio Chi-Square	df	Sig.
2345.694	5	.000

Dependent Variable: Total_HSC

Model: (Intercept), Age, Gender_1st_participant, N_conditions, quint_imd, undiagnosed_based_on_moderat

a. Compares the fitted model against the intercept-only model.

Tests of Model Effects

Source	Type III		
	Wald Chi-Square	df	Sig.

**(Intercept)**	**55.493**	**1**	**<.001**
**Age**	**1.898**	**1**	**.168**
**Gender_1st_participant**	**62.039**	**1**	**<.001**
**N_conditions**	**1382.705**	**1**	**.000**
**quint_imd**	**47.491**	**1**	**<.001**
**undiagnosed_based_on_moderat**	**37.849**	**1**	**<.001**

Dependent Variable: Total_HSC

Model: (Intercept), Age, Gender_1st_participant, N_conditions, quint_imd, undiagnosed_based_on_moderat

Parameter Estimates

			**95% Wald Confidence Interval**		**Hypothesis Test**		
**Parameter**	**B**	**Std. Error**	**Lower**	**Upper**	**Wald Chi-Square**	**df**	**Sig.**
**(Intercept)**	**372.409**	**49.9920**	**274.426**	**470.391**	**55.493**	**1**	**<.001**
**Age**	**-.640**	**.4644**	**-1.550**	**.270**	**1.898**	**1**	**.168**
**Gender_1st_participant**	**132.631**	**16.8390**	**99.628**	**165.635**	**62.039**	**1**	**<.001**
**N_conditions**	**509.686**	**13.7069**	**482.821**	**536.551**	**1382.705**	**1**	**.000**
**quint_imd**	**-56.997**	**8.2707**	**-73.207**	**-40.786**	**47.491**	**1**	**<.001**
**undiagnosed_based_on_moderat**	**250.260**	**40.6785**	**170.532**	**329.989**	**37.849**	**1**	**<.001**

**(Scale)**	**42.953[a]**	**.5080**	**41.969**	**43.960**			

Dependent Variable: Total_HSC

Model: (Intercept), Age, Gender_1st_participant, N_conditions, quint_imd, undiagnosed_based_on_moderat

a. Maximum likelihood estimate.

3. REGRESSION WITHOUT NUMBER OF CONDITIONS OR UNDIAGNOSED MENTAL HEALTH TO EXPLORE IMPACT OF IMD QUINTILE ON COSTS

Generalized Linear Models

Notes

**Output Created**		**17-JAN-2023 14:21:29**
**Comments**		
**Input**	**Data**	**C:\Users\brenc\Documents\work\hhs paper\wave12_withgeo_CG from M drive.sav**

	**Active Dataset**	**DataSet1**
	**Filter**	**<none>**
	**Weight**	**<none>**
	**Split File**	**<none>**
	**N of Rows in Working Data File**	**7731**
**Missing Value Handling**	**Definition of Missing**	**User-defined missing values for factor, subject and within-subject variables are treated as missing.**
	**Cases Used**	**Statistics are based on cases with valid data for all variables in the model.**
**Weight Handling**		**not applicable**

**Syntax**	**GENLIN USE_Total_health_social_care_costs WITH Age Gender_1st_participant N_conditions quint_imd**<br><br>**undiagnosed_MH_use**<br><br>**/MODEL Age Gender_1st_participant N_conditions quint_imd undiagnosed_MH_use INTERCEPT=YES**<br><br>**SCALEWEIGHT=WEIGHT5**<br><br>**DISTRIBUTION=TWEEDIE(1.5) LINK=IDENTITY**<br><br>**/CRITERIA METHOD=FISHER SCALE=MLE COVB=MODEL MAXITERATIONS=50000 MAXSTEPHALVING=10**<br><br>**PCONVERGE=1E-006(ABSOLUTE) SINGULAR=1E-012 ANALYSISTYPE=3(WALD) CILEVEL=95 CITYPE=WALD**<br><br>**LIKELIHOOD=FULL**<br><br>**/MISSING CLASSMISSING=EXCLUDE**<br><br>**/PRINT CPS DESCRIPTIVES MODELINFO FIT SUMMARY SOLUTION.**

Resources	Processor Time	00:00:02.66
	Elapsed Time	00:00:02.66

Warnings

The maximum number of step-halvings was reached but the log-likelihood value cannot be further improved. Output for the last iteration is displayed.
The GENLIN procedure continues despite the above warning(s). Subsequent results shown are based on the last iteration. Validity of the model fit is uncertain.

Model Information

Dependent Variable	Total_HSC
Probability Distribution	Tweedie (1.5)
Link Function	Identity
Scale Weight Variable	WEIGHT5

Case Processing Summary

	N	Percent
Included	6985	90.4%

**Excluded**	**746**	**9.6%**
**Total**	**7731**	**100.0%**

Continuous Variable Information

		N	Minimum	Maximum	Mean	Std. Deviation
Dependent Variable	Total_HSC	6985	.00	203754.00	1000.4431	4068.14276
Covariate	Age	6985	18.00	100.00	49.2278	19.02923
	Gender_1st_participant	6985	1.00	2.00	1.5621	.49617
	N_conditions	6985	0	16	1.47	1.919
	quint_imd	6985	1	5	4.47	1.032
	undiagnosed_MH_use	6985	.00	1.00	.2677	.44280
Scale Weight	WEIGHT5	6985	0	10	.9972	.73440

Goodness of Fit[a]

	Value	df	Value/df
Deviance	505047.681	6979	72.367
Scaled Deviance	12268.813	6979	
Pearson Chi-Square	3429081.329	6979	491.343
Scaled Pearson Chi-Square	83300.564	6979	

Log Likelihood[b]	-50525.297		
Akaike's Information Criterion (AIC)	101064.594		
Finite Sample Corrected AIC (AICC)	101064.610		
Bayesian Information Criterion (BIC)	101112.554		
Consistent AIC (CAIC)	101119.554		

Dependent Variable: Total_HSC

Model: (Intercept), Age, Gender_1st_participant, N_conditions, quint_imd, undiagnosed_MH_use

a. Information criteria are in smaller-is-better form.

b. The full log likelihood function is displayed and used in computing information criteria.

Omnibus Test[a]

Likelihood Ratio Chi-Square	df	Sig.
2862.209	5	.000

Dependent Variable: Total_HSC

Model: (Intercept), Age, Gender_1st_participant, N_conditions, quint_imd, undiagnosed_MH_use

a. Compares the fitted model against the intercept-only model.

Tests of Model Effects

Source	Type III Wald Chi-Square	df	Sig.
(Intercept)	63.050	1	<.001
Age	2.916	1	.088
Gender_1st_participant	75.972	1	.000
N_conditions	1650.578	1	.000
quint_imd	63.203	1	<.001
undiagnosed_MH_use	129.585	1	.000

Dependent Variable: Total_HSC

Model: (Intercept), Age, Gender_1st_participant, N_conditions, quint_imd, undiagnosed_MH_use

Parameter Estimates

			95% Wald Confidence Interval		Hypothesis Test		
Parameter	B	Std. Error	Lower	Upper	Wald Chi-Square	df	Sig.
(Intercept)	358.206	45.1119	269.789	446.624	63.050	1	<.001

Age	-.677	.3963	-1.453	.100	2.916	1	.088
Gender_1st_participant	128.717	14.7676	99.773	157.661	75.972	1	.000
N_conditions	492.692	12.1271	468.923	516.461	1650.578	1	.000
quint_imd	-60.573	7.6192	-75.506	-45.639	63.203	1	<.001
undiagnosed_MH_use	253.380	22.2584	209.754	297.006	129.585	1	.000
(Scale)	41.165[a]	.4522	40.288	42.061			

Dependent Variable: Total_HSC

Model: (Intercept), Age, Gender_1st_participant, N_conditions, quint_imd, undiagnosed_MH_use

a. Maximum likelihood estimate.

4. REGRESSION OF EQ-5D INDEX SCORE WITHOUT UNDIAGNOSED MENTAL HEALTH

```
REGRESSION
  /MISSING LISTWISE
  /REGWGT=WEIGHT5
  /STATISTICS COEFF OUTS R ANOVA
  /CRITERIA=PIN(.05) POUT(.10)
  /NOORIGIN
```

```
/DEPENDENT EQ5D_utility
/METHOD=ENTER Age Gender_1st_participant N_conditions quint_imd
/RESIDUALS NORMPROB(ZRESID).
```

Regression

Notes

Output Created		11-DEC-2019 15:44:49
Comments		
Input	Data	M:\HHS work\wave12_withgeo_CG.sav
	Active Dataset	DataSet1
	Filter	New_or_repeat=1 (FILTER)
	Weight	<none>
	Split File	<none>
	N of Rows in Working Data File	6675
Missing Value Handling	Definition of Missing	User-defined missing values are treated as missing.

	Cases Used	Statistics are based on cases with no missing values for any variable used.
Syntax		REGRESSION<br>/MISSING LISTWISE<br>/REGWGT=WEIGHT5<br>/STATISTICS COEFF OUTS R ANOVA<br>/CRITERIA=PIN(.05) POUT(.10)<br>/NOORIGIN<br>/DEPENDENT EQ5D_utility<br>/METHOD=ENTER Age Gender_1st_participant N_conditions quint_imd<br>/RESIDUALS NORMPROB(ZRESID).
Resources	Processor Time	00:00:00.09
	Elapsed Time	00:00:00.09
	Memory Required	47552 bytes
	Additional Memory Required for Residual Plots	248 bytes

Warnings

No plots are produced for Weighted Least Squares regression. You can SAVE the appropriate variables and use other procedures (e.g., EXAMINE and PLOT) to produce the requested plots. To plot weighted versions of the residuals and predicted values, use COMPUTE before plotting: COMPUTE RESID = SQRT(REGWGTvar) * RESID COMPUTE PRED = SQRT(REGWGTvar) * PRED.

Variables Entered/Removed[a,b]

Model	Variables Entered	Variables Removed	Method
1	quint_imd, Gender_1st_participant, N_conditions, Age[c]	.	Enter

a. Dependent Variable: EQ5D_utility

b. Weighted Least Squares Regression - Weighted by WEIGHT5

c. All requested variables entered.

Model Summary[b,c]

Model	R	R Square	Adjusted R Square	Std. Error of the Estimate
1	.615[a]	.378	.378	.2264322

a. Predictors: (Constant), quint_imd, Gender_1st_participant, N_conditions, Age

b. Dependent Variable: EQ5D_utility

c. Weighted Least Squares Regression - Weighted by WEIGHT5

ANOVA[a,b]

Model		Sum of Squares	df	Mean Square	F	Sig.
1	Regression	186.525	4	46.631	909.498	.000[c]
	Residual	306.604	5980	.051		
	Total	493.129	5984			

a. Dependent Variable: EQ5D_utility

b. Weighted Least Squares Regression - Weighted by WEIGHT5

c. Predictors: (Constant), quint_imd, Gender_1st_participant, N_conditions, Age

Coefficients[a,b]

Model		Unstandardized Coefficients		Standardized Coefficients	t	Sig.
		B	Std. Error	Beta		
1	(Constant)	1.134	.016		69.379	.000
	Age	-.001	.000	-.076	-6.924	.000

	Gender_1st_participant	-.012	.006	-.021	-2.034	.042
	N_conditions	-.095	.002	-.567	-51.498	.000
	quint_imd	-.028	.003	-.113	-10.998	.000

a. Dependent Variable: EQ5D_utility

b. Weighted Least Squares Regression - Weighted by WEIGHT5

Residuals Statistics[a,b]

	Minimum	Maximum	Mean	Std. Deviation	N
Predicted Value	-.630765	1.073533	.798142	.1933642	5985
Residual	-1.3520548	1.1467655	-.0130610	.2524559	5985
Std. Predicted Value[c]	.	.	.	.	0
Std. Residual[c]	.	.	.	.	0

a. Dependent Variable: EQ5D_utility

b. Weighted Least Squares Regression - Weighted by WEIGHT5

c. Not computed for Weighted Least Squares regression.

5. REGRESSION OF EQ-5D INDEX SCORE WITH UNDIAGNOSED MENTAL HEALTH

```
REGRESSION
  /MISSING LISTWISE
  /REGWGT=WEIGHT5
  /STATISTICS COEFF OUTS R ANOVA
  /CRITERIA=PIN(.05) POUT(.10)
  /NOORIGIN
  /DEPENDENT EQ5D_utility
  /METHOD=ENTER Age Gender_1st_participant N_conditions quint_imd undiagnosed_MH_use
  /RESIDUALS NORMPROB(ZRESID).
```

Regression

Notes

Output Created		11-DEC-2019 15:45:43
Comments		
Input	Data	M:\HHS work\wave12_withgeo_CG.sav

	Active Dataset	DataSet1
	Filter	New_or_repeat=1 (FILTER)
	Weight	<none>
	Split File	<none>
	N of Rows in Working Data File	6675
Missing Value Handling	Definition of Missing	User-defined missing values are treated as missing.
	Cases Used	Statistics are based on cases with no missing values for any variable used.
Syntax		REGRESSION<br>/MISSING LISTWISE<br>/REGWGT=WEIGHT5<br>/STATISTICS COEFF OUTS R ANOVA<br>/CRITERIA=PIN(.05) POUT(.10)<br>/NOORIGIN<br>/DEPENDENT EQ5D_utility<br>/METHOD=ENTER Age Gender_1st_participant N_conditions quint_imd undiagnosed_MH_use<br>/RESIDUALS NORMPROB(ZRESID).

Resources	Processor Time	00:00:00.11
	Elapsed Time	00:00:00.09
	Memory Required	48208 bytes
	Additional Memory Required for Residual Plots	232 bytes

Warnings

No plots are produced for Weighted Least Squares regression. You can SAVE the appropriate variables and use other procedures (e.g., EXAMINE and PLOT) to produce the requested plots. To plot weighted versions of the residuals and predicted values, use COMPUTE before plotting: COMPUTE RESID = SQRT(REGWGTvar) * RESID COMPUTE PRED = SQRT(REGWGTvar) * PRED.

Variables Entered/Removed[a,b]

Model	Variables Entered	Variables Removed	Method
1	undiagnosed_MH_use, Age, Gender_1st_participant, quint_imd, N_conditions[c]	.	Enter

a. Dependent Variable: EQ5D_utility

b. Weighted Least Squares Regression - Weighted by WEIGHT5

c. All requested variables entered.

Model Summary[b,c]

Model	R	R Square	Adjusted R Square	Std. Error of the Estimate
1	.618[a]	.382	.382	.2257455

a. Predictors: (Constant), undiagnosed_MH_use, Age, Gender_1st_participant, quint_imd, N_conditions

b. Dependent Variable: EQ5D_utility

c. Weighted Least Squares Regression - Weighted by WEIGHT5

ANOVA[a,b]

Model		Sum of Squares	df	Mean Square	F	Sig.
1	Regression	188.433	5	37.687	739.519	.000[c]
	Residual	304.696	5979	.051		
	Total	493.129	5984			

a. Dependent Variable: EQ5D_utility

b. Weighted Least Squares Regression - Weighted by WEIGHT5

c. Predictors: (Constant), undiagnosed_MH_use, Age, Gender_1st_participant, quint_imd, N_conditions

Coefficients[a,b]

Model		Unstandardized Coefficients		Standardized Coefficients	t	Sig.
		B	Std. Error	Beta		
1	(Constant)	1.140	.016		69.837	.000
	Age	-.001	.000	-.076	-6.949	.000
	Gender_1st_participant	-.011	.006	-.020	-1.954	.051
	N_conditions	-.095	.002	-.565	-51.465	.000
	quint_imd	-.027	.003	-.110	-10.700	.000
	undiagnosed_MH_use	-.040	.007	-.062	-6.119	.000

a. Dependent Variable: EQ5D_utility

b. Weighted Least Squares Regression - Weighted by WEIGHT5

Residuals Statistics[a,b]

	Minimum	Maximum	Mean	Std. Deviation	N
Predicted Value	-.654617	1.081059	.798473	.1942564	5985
Residual	-1.3230425	1.1706175	-.0133921	.2517701	5985
Std. Predicted Value[c]	.	.	.	.	0
Std. Residual[c]	.	.	.	.	0

a. Dependent Variable: EQ5D_utility

b. Weighted Least Squares Regression - Weighted by WEIGHT5

c. Not computed for Weighted Least Squares regression.

6. REGRESSION WITH UNDIAGNOSED MENTAL HEALTH BASED ON MODERATE CUTOFF FOR PHQ9 AND GAD7

Variables Entered/Removed[a,b]

Model	Variables Entered	Variables Removed	Method
1	undiagnosed_based_on_moderat, Gender_1st_participant, Age, quint_imd, N_conditions[c]	.	Enter

a. Dependent Variable: EQ5D_utility

b. Weighted Least Squares Regression - Weighted by WEIGHT5

c. All requested variables entered.

Model Summary[b,c]

Model	R	R Square	Adjusted R Square	Std. Error of the Estimate
1	.620[a]	.385	.384	.2252517

a. Predictors: (Constant), undiagnosed_based_on_moderat, Gender_1st_participant, Age, quint_imd, N_conditions

b. Dependent Variable: EQ5D_utility

c. Weighted Least Squares Regression - Weighted by WEIGHT5

ANOVA[a,b]

Model		Sum of Squares	df	Mean Square	F	Sig.
1	Regression	189.765	5	37.953	748.014	.000[c]
	Residual	303.364	5979	.051		
	Total	493.129	5984			

a. Dependent Variable: EQ5D_utility

b. Weighted Least Squares Regression - Weighted by WEIGHT5

c. Predictors: (Constant), undiagnosed_based_on_moderat, Gender_1st_participant, Age, quint_imd, N_conditions

Coefficients[a,b]

		Unstandardized Coefficients		Standardized Coefficients		
Model		B	Std. Error	Beta	t	Sig.
1	(Constant)	1.140	.016		70.047	.000
	Age	-.001	.000	-.081	-7.383	<.001
	Gender_1st_participant	-.012	.006	-.021	-2.021	.043
	N_conditions	-.094	.002	-.560	-50.943	.000
	quint_imd	-.027	.003	-.110	-10.738	<.001

	undiagnosed_based_on_moderat	-.076	.010	-.081	-7.990	<.001

a. Dependent Variable: EQ5D_utility

b. Weighted Least Squares Regression - Weighted by WEIGHT5

Residuals Statistics[a,b]

	Minimum	Maximum	Mean	Std. Deviation	N
Predicted Value	-.682313	1.079419	.798317	.1946455	5985
Residual	-1.3622371	1.1983134	-.0132355	.2512853	5985
Std. Predicted Value[c]	.	.	.	.	0
Std. Residual[c]	.	.	.	.	0

a. Dependent Variable: EQ5D_utility

b. Weighted Least Squares Regression - Weighted by WEIGHT5

c. Not computed for Weighted Least Squares regression.

7.
